# A little (up)SET THEORY: a philosophical and psychological pondering of a scientist on the state of our art

**DOI:** 10.1186/s40697-015-0084-3

**Published:** 2015-12-01

**Authors:** András Kapus

**Affiliations:** Keenan Research Centre for Biomedical Science of St. Michael’s Hospital, Room 621, 209 Victoria Street, Toronto, ON M5B 1T8 Canada; Department of Surgery and Department of Biochemistry, University of Toronto, Toronto, ON M5B 1T8 Canada

Ever since Thomas Kuhn’s brilliant postulate that scientific progress is generated in the context of paradigms (commonly accepted notions), and that scientific revolutions manifest as paradigm shifts, we not only work along paradigms but are striving to consciously create them. Moreover, paradigms emerge not only *within* science but also *about* science, i.e., paradigms about how science should be done and how it should interact with society. In our field, the currently reigning paradigm (or more properly perhaps meta-paradigm or zeitgeist) is undoubtedly that of knowledge translation. Indeed, knowledge translation (KT) is a crucially important concept. And yet when a paradigm or rather its application becomes a self-imposed dogma or an overbearing cliché, it may counteract progress.

When one receives criticism that one’s work is “cutting edge research in an enigmatic area in cell biology but the explanation how it is translational to clinic is weak,” one starts pondering the zeitgeist that compels referees to assess everything from the KT angle. And during such pondering over the current state of affairs, it suddenly dawned on me that the very concept of knowledge translation *as it is applied today*, is fundamentally—philosophically—flawed. In fact, it is a misnomer. What is being forced on us is not knowledge translation but *science translation.* And of course, knowledge and science are not the same. Far from it. Let me explain.

## Knowledge translation vs. science translation—a culture reduced to the limited terms of capitalism

If you know how to make excellent “al dente” pasta, that is great knowledge. But that is not science. If you have a sparkling idea how a process might work and some murky thoughts about how to test that—that is science but not knowledge. Science and knowledge are two separate sets, with a substantial overlap. In fact, some elements of the Science set (call it S) will become elements of the Knowledge set (K), and then some elements of the Knowledge set can be translated to elements of Practical use (P). But S is not equal to K (S ≠ K), and S cannot be directly translated to P. Further, there are elements of K, which never have been science, that can be readily translated into useful application. Importantly, by not supporting S, we will not only empty K but will also actively inhibit both the S → K and the K → P transitions because science itself finds ways to facilitate these processes.

Science is a *culture* and a way of thinking; knowledge is an ordered store of (potentially usable) information. There is nothing wrong with knowledge translation per se, which is undoubtedly a fundamental component of the impact of science on society. However, the dramatic and misleading mental lapse that calls for knowledge translation (KT) but in fact demands science translation (ST) may be an important manifestation of the most dangerous unfolding drama of the information age. Namely, the thought that the concepts of capitalistic production (efficiency, profit, marketability, commercialization, media attention, patent, spin-off company, etc.) should be directly applicable to science (and should be the gold standard with which we measure the value of research!). In other words, the current zeitgeist demands that there be a one-to-one correspondence (to use a basic concept of set theory) between the elements of P and S. Moreover, the demanded correspondence is backward (P → S), so the elements of P should generate the elements of S and justify their value. Again, there is nothing wrong with knowledge translation per se; but it should not be forged to mean science translation (ST is a non-existing concept), should not be forced upon all scientific approaches (as it is the current practice in life sciences), and its subsidization should not jeopardize the support for basic scientific discovery.

The dangers of the current, direct profit-oriented view are manifold, far-reaching, and profound; they are particularly frightening because in our business-centered society, this profit-driven worldview has taken the guise of a self-evident truth. But our contemporary business-minded model is misleading and is not supported by the history of scientific progress; therefore, we scientists are increasingly responsible for informing the public about the mechanisms through which science has generated real breakthroughs. The current dominant view does not tell the real story of how curiosity did not kill the cat but *cured the cat.* Selecting one of thousands of examples, if Akira Endo had not asked why certain mushrooms are so good at killing their parasites, we would not have statins, which have been shown to reduce cardiovascular death by 30 % (and which might save us from a stroke at the next grant rejection). But seriously, the narrow-minded application of the current approach is a major *disservice to society*, slowing or diverting the very process which leads to real and translatable solutions to many of the world’s burning problems, a fact made all the more tragic since we live in a time when we have a real chance to achieve these goals. And let me add one more shade to these complexities. There are (were) initiatives which served truly meaningful forms of knowledge translation. One of these was the MD/PhD program, which equipped the doctors of the future with strong training in fundamental science and basic research, allowing them to speak the languages of both theory and practice (and thereby truly translate between these), making them capable of appreciating, generating, and applying science. The recent cancelation of federal support for this program is as irrational and irresponsible as it is hypocritical.

*What to fund* is a key question, *how to fund it* is another. Clearly, the absence of predictability of funding decisions (e.g., the uncoupling of good, peer-reviewed scientific output from continued support) together with chronic underfunding has generated an emergent state that can be called the “negative nitrogen balance in research.” Insufficient protein intake results in net protein loss, and hence, negative nitrogen balance. This exact parallel is found in research where insufficient support not only halts progress but generates net losses in terms of termination of highly qualified personnel (e.g., when our best and most productive and knowledgeable technicians become unaffordable), disposal of precious tools (e.g., animal and yeast colonies, libraries, etc.), and huge stretches of time and effort (e.g., the generation and regeneration of never-finalized and therefore perpetually preliminary data for grant applications). Thus, ironically, a system, which puts competitive efficiency on its banner, becomes increasingly inefficient and, to elaborate the metaphor, malnourished.

## The impact of the system on the spirit of inquiry and on the inquiring spirit

And we have not even taken into account the psychology of science-making yet. Where is the evidence proving that the state of being continuously threatened, living under the unrelenting pressure to align and comply, the burgeoning formalism, and all-pervasive competitiveness are the most efficient ways (borrowing again a concept from the production line) to do science and—as a consequence—to generate knowledge? While toughness and endurance are inevitably necessary—as nature is secretive enough—do these additional artificial hurdles not choke or scare away many of the most sensitive and original minds who are just not cut out for this propaganda-embellished, distorted struggle? Will we not lose those who have become scientists for the love of findings things out (as Feynman puts it) and not for advertising themselves and selling products? Are we not contra-selecting? Are we not losing the beauty in the beast? Do we not convert (quite often) burning enthusiasm to burnt-out enthusiasts? The current trend does not favor or often even allow risk taking, daydreaming, contemplation and failure, all of which are essential components of any creative process. In fact, productivity (a concept of business mentality) is much favored over creativity (a concept of scientific mentality). Society advocates multiculturalism over monolithic dogmas; why then in science, one of the highest domains of human culture, have we become so narrow-minded and dogmatic? How has *a sense of relevance* become more important and more valued than the pursuit of in-depth understanding?

Lately, I have also noticed some alarming signs in ourselves, in the way we turn to scientific questions—trends that involve what I call *self-censorship* and *thought abortion*. We gradually cease to see things for what they are, viewing them in the false light of what they might (or should) become, what they could be used for—a paradox, since their full potential will never surface unless we uncover their true nature. In the past, when I saw an interesting phenomenon, my question was “how does it come about, what could be the mechanism of it?” Nowadays, the question is “how it can be pragmatically applied, what it is good for?” And if the answer is not immediately clear (and how would it be without having the knowledge?), then the following stereotype sets in: I cannot propose to investigate this process in a grant because nobody will accept it as a “legitimate” question. Or I should come up with an (often terribly overstretched) application just to justify the question. One knows that a straightforward approach just will not work in the current funding system whose philosophy (rather amusingly) regards basic science as “l’art pour l’art"t. So asking silly questions—such as how come that bacteria can grow in hot springs or why petunias suddenly lose their color—enquiries that gave us PCR and the siRNA technology—has become obsolete. Thus, we do not dare to ask or pursue such childish thoughts from which—obviously—neither society nor the enquirer will benefit. Indeed, such naïve ambitions are efficiently purged from the psyche of the realistic scientist. But do we not sacrifice reality for the sake of being realistic?

I know the power of slogans in human history. I grew up in an East-European society that advertised itself with banners saying “Face the Railways” and “With the People through Fire and Water.” Slogans rarely promoted the well-being of this planet. Culture has thoughts and not slogans. It strives for understanding not for clichés. Everybody pays lip service to out-of-the-box thinking, but the “boxiness” of our age has only grown. It is now the out-of-the-box box. Let us leave the adjectives. Let us just return to thinking. Let us protect our productive paradigms from becoming detrimental dogmas. A better, sustainable balance will alleviate my upSET THEORY.

